# Bursa-Derived Cells Show a Distinct Mechano-Response to Physiological and Pathological Loading *in vitro*

**DOI:** 10.3389/fcell.2021.657166

**Published:** 2021-05-31

**Authors:** Franka Klatte-Schulz, Nicole Bormann, Isabel Voss, Josephine Melzer, Aysha Schmock, Christian H. Bucher, Kathi Thiele, Philipp Moroder, Melanie Haffner-Luntzer, Anita Ignatius, Georg N. Duda, Britt Wildemann

**Affiliations:** ^1^Julius Wolff Institute, Berlin Institute of Health at Charité – Universitätsmedizin Berlin, Berlin, Germany; ^2^BIH Center for Regenerative Therapies (BCRT), Berlin Institute of Health at Charité – Universitätsmedizin Berlin, Berlin, Germany; ^3^Center for Musculoskeletal Surgery, Charité – Universitätsmedizin Berlin, Berlin, Germany; ^4^Institute of Orthopaedic Research and Biomechanics, Ulm University, Ulm, Germany; ^5^Experimental Trauma Surgery, Department of Trauma-, Hand- and Reconstructive Surgery, Jena University Hospital, Friedrich Schiller University Jena, Jena, Germany

**Keywords:** subacromial bursa, bursa-derived cells, mechanical stimulation, mechano-transduction, matrix remodeling

## Abstract

The mechano-response of highly loaded tissues such as bones or tendons is well investigated, but knowledge regarding the mechano-responsiveness of adjacent tissues such as the subacromial bursa is missing. For a better understanding of the physiological role of the bursa as a friction-reducing structure in the joint, the study aimed to analyze whether and how bursa-derived cells respond to physiological and pathological mechanical loading. This might help to overcome some of the controversies in the field regarding the role of the bursa in the development and healing of shoulder pathologies. Cells of six donors seeded on collagen-coated silicon dishes were stimulated over 3 days for 1 or 4 h with 1, 5, or 10% strain. Orientation of the actin cytoskeleton, YAP nuclear translocation, and activation of non-muscle myosin II (NMM-II) were evaluated for 4 h stimulations to get a deeper insight into mechano-transduction processes. To investigate the potential of bursa-derived cells to adapt their matrix formation and remodeling according to mechanical loading, outcome measures included cell viability, gene expression of extracellular matrix and remodeling markers, and protein secretions. The orientation angle of the actin cytoskeleton increased toward a more perpendicular direction with increased loading and lowest variations for the 5% loading group. With 10% tension load, cells were visibly stressed, indicated by loss in actin density and slightly reduced cell viability. A significantly increased YAP nuclear translocation occurred for the 1% loading group with a similar trend for the 5% group. NMM-II activation was weak for all stimulation conditions. On the gene expression level, only the expression of TIMP2 was down-regulated in the 1 h group compared to control. On the protein level, collagen type I and MMP2 increased with higher/longer straining, respectively, whereas TIMP1 secretion was reduced, resulting in an MMP/TIMP imbalance. In conclusion, this study documents for the first time a clear mechano-responsiveness in bursa-derived cells with activation of mechano-transduction pathways and thus hint to a physiological function of mechanical loading in bursa-derived cells. This study represents the basis for further investigations, which might lead to improved treatment options of subacromial bursa-related pathologies in the future.

## Introduction

Mechanical stimuli are known to be sensed especially by cells of mechanically highly loaded tissues, which are directly responsible for movement such as bones, tendons, and muscles. For adjacent tissues that are only indirectly loaded, such mechano-responsiveness has so far not been investigated. One of these adjacent tissues is the subacromial bursa, which is responsible for reducing friction between the rotator cuff tendons and the acromion at the shoulder (Draghi et al., 2015). Knowledge about the mechano-responsiveness of bursa-derived cells is needed to better understand the physiological function of the subacromial bursa as a friction-reducing structure in the shoulder joint. The subacromial bursa is often related to shoulder pain and restricted movement due to bursal inflammation. Therefore, the removal of the bursa is an often performed surgical procedure. However, there exist currently several controversies in the field regarding the effectiveness of this procedure (Butt et al., 2015; Beard et al., 2017; Farfaras et al., 2018). It remains unclear if the subacromial bursa might also have a regenerative capacity for surrounding tissues as it contains high amounts of growth factors, is highly vascularized, and also contains stem cells (Steinert et al., 2015; Chillemi et al., 2016; Poldoja et al., 2017). This underlines the fact that the role of the subacromial bursa in the shoulder joint and its impact on the development or healing of shoulder pathologies are still unclear. The aim of the present study is to obtain a more detailed picture of the subacromial bursa by understanding the role of mechanical strain on bursa-derived cells.

Several *in vitro* studies have been published that investigate the effect of mechanical stimulation on different cell types of the musculoskeletal system that are known to be highly loaded and in consequence their mechano-responsiveness was analyzed using different loading devices. As summarized by Wang et al., for tendon-derived cells, it was shown that in 2D uniaxial and biaxial stimulation, high loading magnitudes lead to increased apoptosis, expression of inflammatory markers, and matrix-degrading enzymes [matrix metalloproteinases (MMPs)]. On the contrary, physiological loading was described to induce rather anabolic effects, such as increased collagen expression (Wang et al., 2018). However, little is known on mechano-transduction of cells adjacent to such highly loaded tissues and whether these would also adapt their extracellular matrix (ECM) formation and remodeling or cell metabolism due to different mechanical stresses. In particular, the bursa-derived cells, which have a fibroblast-like, spindle-shaped morphology and a clear stem cell potential (Steinert et al., 2015; Morikawa et al., 2019), are of interest since the bursa is next to the direct mechanically loaded structures of the shoulder an often target for surgical treatment. However, it is so far unknown, if bursa-derived cells are able to change the composition of the ECM and, with this, the friction-reducing capacity of the bursa.

Mechano-transduction is the process by which cells actively sense and convert mechanical stimuli into biomechanical signals resulting in intracellular changes. These cellular responses to mechanical stimuli are highly variable and include changes in ion concentration, activation of signaling pathways, and transcriptional regulations. The Yes-associated protein (YAP) is an important regulator of various cellular processes such as proliferation, differentiation, and survival. Together with the transcriptional coactivator with PDZ-binding motif (TAZ), YAP has first been described as an important mechano-transducer (Dupont et al., 2011). Since then, it has been shown in several studies that YAP/TAZ are regulated in response to many different mechanical stimuli as summarized by Dobrokhotov et al. (2018). YAP/TAZ are activated through phosphorylation and nuclear translocation, which is induced by tension of the actin cytoskeleton leading to widening of nuclear pores finally resulting in increased YAP import (Elosegui-Artola et al., 2017). YAP activation is highly dependent on the ECM stiffness (Dupont et al., 2011), which in turn is regulated by the composition of ECM components such as collagens and proteoglycans. Non-muscle myosin II (NMM-II) is an actin binding motor protein, which has an important function in cell proliferation, migration, and adhesion (Vicente-Manzanares et al., 2009; Lu et al., 2020). Furthermore, NMM-II is essential for ECM stiffness generation and for sensing of matrix and shear stresses (Shin et al., 2013). The activity of NMM-II depends on the phosphorylation of the myosin regulatory light chain (MLC), which initiates myosin ATPase activity (Vicente-Manzanares et al., 2009). In the current study, the YAP/TAZ and NMM-II pathways are investigated to prove that the response of bursa-derived cells to adapt their ECM production/remodeling is activated through the ability of the cells to actively sense mechanical stimuli.

When investigating the response of cells to mechanical loading regarding matrix and cellular adaptation, several components are of interest. Integrins link the ECM to the actin cytoskeleton of the cell as transmembrane receptors. They are key mediators in the transduction of mechanical loading from the matrix to the nuclear processes. Their regulation was previously shown in different *in vitro* loading models (Kaneko et al., 2014; Popov et al., 2015). Consequently, the regulation of ECM components such as collagens and proteoglycans is important in loaded cells. Fibromodulin regulates the collagen fiber arrangement as well as the collagen fibril diameter and therefore organizes the ECM composition (Jan et al., 2016; Kalamajski et al., 2016). Versican ensures an increased pressure resistance under load by increasing the water content in the affected regions (Vogel, 2004). In addition to collagen type I and III (Col I/III), the regulation of Fibromodulin and Versican expression was previously shown in mechanically stretched tendon progenitor cells (Popov et al., 2015). MMPs and their natural inhibitors, the tissue inhibitors of metalloproteinases (TIMPs), regulate the modeling and remodeling of various tissues and the balance between MMPs and TIMPs is important to maintain tissue homeostasis (Loffek et al., 2011). MMPs are downstream targets of integrins, and their expression is regulated upon mechanical stimulation, as demonstrated in *in vitro* studies with different musculoskeletal cell types (Fujisawa et al., 1999; Yang et al., 2005; Sasaki et al., 2007). Investigating the relevance of different mechanical loading regimes on the ECM formation and remodeling is important to understand the adaptation of the bursa to mechanical stress conditions.

The present study aimed to improve the understanding of the physiological role of the subacromial bursa as a friction-reducing tissue, by investigating whether and how bursa-derived cells respond to mechanical strain by adapting the matrix formation and remodeling. Furthermore, we aim to detect possible physiological and pathological limits of this mechanical response. Currently, bursa tissue is widely seen as a passive structure of the shoulder joint. With the knowledge that different mechanical strain signals can actively be sensed by these cells and transduced into an intracellular response, a novel interpretation of this tissue might be necessary and eventually lead to improving current treatment strategies of subacromial bursa-related pathologies.

Low (1%), medium (5%), and high (10%) strain magnitudes have been used in a modified mechanical loading device (Neidlinger-Wilke et al., 1994). To confirm mechano-transduction processes in these cells, YAP nuclear translocation and the activation of NMM-II were evaluated. As readouts of cellular mechano-response, key parameters included cell viability, orientation of the cytoskeleton, gene expression of markers associated with ECM formation and remodeling (Col I and III, Versican, Fibromodulin, MMPs, and TIMPs), as well as partially respective protein secretion.

## Materials and Methods

### Isolation of Bursa-Derived Cells

Bursa tissue was harvested from six donors (two females and four males, mean age: 69.2 ± 9.1 years) during open surgery according to glenohumeral pathologies such as humeral head necrosis or omarthrosis. All patients have been treated for persisting chronic pathologies of the shoulder. The harvesting and use of bursa tissue were approved by the local IRB (EA1/267/15). The bursa tissue was chopped and digested in 0.3% collagenase type II solution (Biochrom, Berlin, Germany) for 2 h at 37°C to harvest the cells. The digested bursa was washed and plated in a cell culture flask in isolation medium consisting of Dulbecco’s Minimal Essential Medium low glucose (DMEM, Biochrom, Berlin, Germany) supplemented with 20% fetal calf serum superior (FCS, Biochrom, Berlin, Germany) and 1% penicillin/streptomycin (P/S, Biochrom, Berlin, Germany). A first change of medium was performed 4–5 days after seeding and afterward every 2–3 days. Bursa-derived cells were expanded and afterward trypsinized (0.05% trypsin/0.02% EDTA in PBS) and frozen in cryo medium (isolation medium with 10% DMSO) in liquid nitrogen until used for the mechanical stimulation experiments. Characterization of four of the six bursa-derived cell cultures was performed regarding their expression profile of fibroblast-associated markers (Vimentin, S100 calcium-binding protein A4) and ECM markers (Collagen type I, II, III, Aggrecan, Decorin, Versican, and Fibromodulin) using qRT-PCR as described in section “Gene Expression Analysis.” Furthermore, for three bursa-derived cell cultures, the progenitor cell potential according to the minimal mesenchymal stromal cell criteria (Dominici et al., 2006) was investigated as performed previously for tenocytes (Klatte-Schulz et al., 2012, 2013, 2014). Details about the materials and methods are given in Supplementary Material and Supplementary Table 1.

### Mechanical Stretching Device

**FIGURE 1:**
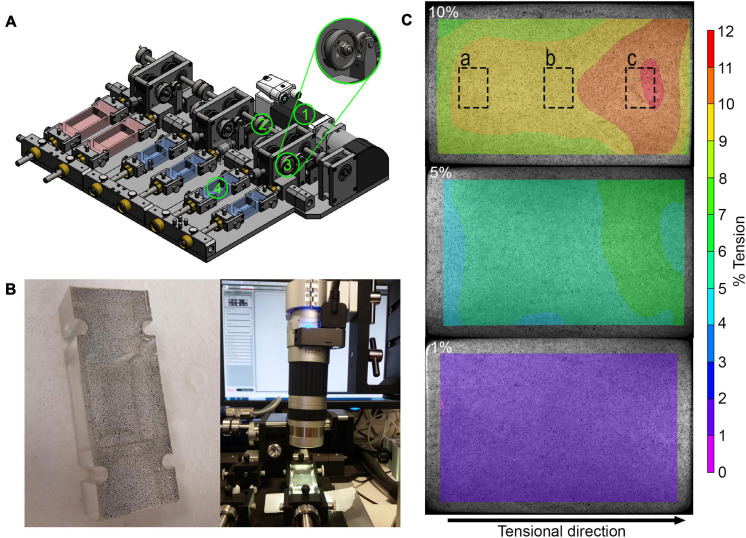
Analyses of mechanical straining in the stretching device: **(A)** Schematic overview of mechanical stretching device. The motor (1) generates a torque, which is transmitted to the rotation axis (2) and then to the eccentric disk (3), which ensures that the flexible silicone dish (4) is stretched. The stretching device also allows to stretch small and large silicon dishes (red). **(B)** Speckled base of the silicon dish (left) and light microscopic evaluation of speckled base of silicon dish (right). **(C)** Exemplary results after speckle analysis of the base at 10, 5, and 1% strain showing strain distribution across the cell dish area. The dashed boxes show the approximate areas where the images were taken for analysis of cell orientation and YAP nuclear translocation.

The mechanical stretching device (Figure 1A) was re-produced by the Center for Scientific Workshops of the Charité - Universitätsmedizin Berlin modifying a stimulation device first published by Neidlinger-Wilke et al. (1994). The stretching device offers the possibility to stretch six silicon dishes in parallel with a surface area of 2 × 3 cm. Up to five different strain magnitudes can be used (1–10% stretch) at strain frequencies of 0.5–2 Hz using a motor (ASB42C048060-ENM, Nanotec Electronic, Feldenkirchen, Germany) and engine control (CANopen C5-E-1-09, Nanotec Electronic) unit.

### Speckle Analysis

To test the accuracy and quality of the stretching, a speckle analysis with digital image correlation was performed. Using an air brush system (Güde, Wolpertshausen, Germany), small speckles were sprayed on the bottom of the silicon dish (Figure 1B). The speckled silicon dish was placed into the dish holder and stretched slowly and manually from minimum to maximum tension while continuous microscopic images were taken during the stretching process using the DVM2500 microscope with a × 0.3 objective and the Leica Application Suite V4.6 software (Leica Microsystems, Wetzlar, Germany). The displacement of the individual speckles was analyzed using the VIC-2D^TM^ 2009 program (Correlated SOLUTIONS, Irmo, SC, United States). The strain magnitude represents the speckle displacement, meaning the distance of speckles at maximum position relative to the distance at the reference position given in %.

### Manufacturing of Silicon Dishes

The silicon ELASTOSIL^®^ RT 601 components A and B (Wacker Chemie, Munich, Germany) were mixed at a ratio of 9 to 1 (w/w) and outgassed using a vacuum pump at 600 mbar. The liquid silicon was poured into the casters using a 20 ml syringe until the caster was filled to the top. The polymerization was done in a heater at 60°C for 1.5 h. Afterward, the hardened silicon was removed from the caster supported by dish wash solution and an ultrasonic bath. Each new silicon dish was pre-conditioned by a three times coating with 50 μg/ml collagen solution [3.56 mg/ml rat tail collagen type I stock solution (Corning, NY, United States) in 0.02% acetic acid] for 24 h at 4°C, to increase the adhesive properties of the silicon surface. Between each coating step, the silicon dishes were cleaned in detergent solution and sterilized in a heat chamber for 2 h at 140°C.

### Mechanical Stimulation of Bursa-Derived Cells

**FIGURE 2:**
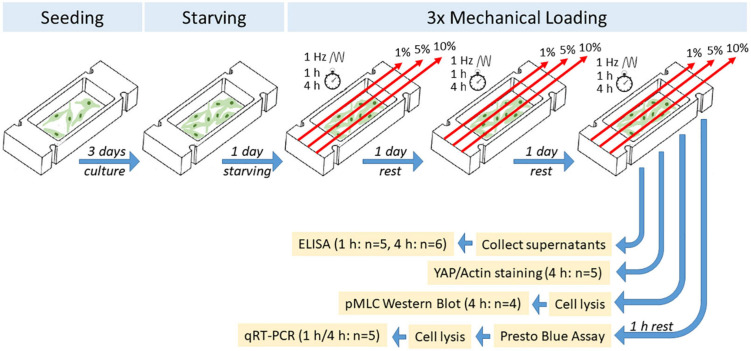
Schematic overview of experimental setup of mechanical loading of bursa-derived cells.

Bursa-derived cells were thawed and expanded in cell culture flasks in growth medium (DMEM low glucose supplemented with 10% FCS superior and 1% P/S). The cells were trypsinized and seeded at passage 2 or 3 with 3 × 10^4^ cells per collagen-coated silicon dish in 2 ml of growth medium. Cells were grown in the dishes for 3 days to about 80% confluence. Afterward, cells were stimulated in stimulation medium (DMEM low glucose, 1% FCS without P/S) on 3 consecutive days at 1 Hz for 1 or 4 h per day at 1, 5, and 10% strain. The stimulation conditions were adapted according to a previous publication using the original stimulation device with tendon progenitor cells (Popov et al., 2015). According to knowledge from tendons, up to 5% strain represents physiological conditions, whereas strains higher than 8% lead to tissue rupture (Arnoczky et al., 2007). Serum reduction was performed to reduce the mitogenic effect of the growth factors in the serum in order to examine only the effect of the mechanical stimulation. Bursa-derived cells grown on silicon dishes without mechanical stimulation served as controls. Cell culture supernatants were collected after stimulation from all donors to analyze the protein synthesis of Col I, MMP1, MMP2, MMP3, and TIMP1. After 1 h of rest, cell viability was measured by PrestoBlue Assay (1:10 in stimulation medium; Invitrogen, Carlsbad, CA, United States) in cells of five donors with an incubation time of 1 h, measured at 560 nm excitation and 590 nm emission (Tecan spectrophotometer Infinite Serie pro200, Tecan Group, Männedorf, Switzerland). Subsequently, RNA was isolated from the bursa-derived cells to investigate regulation on the gene expression level. The resting period aimed to optimally detect adaptations in gene expression after the mechanical stimulation. Bursa-derived cells of five donors stimulated for 4 h were fixed directly after stimulation and stained for actin filaments and YAP. Additionally, cells from four donors were lysed directly after stimulation to evaluate the activation of NMM-II. An overview of the experimental setup is depicted in Figure 2 and methods are described in detail below.

### Orientation of the Cytoskeleton and YAP Nuclear Translocation

Immunofluorescence staining was performed to investigate the orientation of the actin filaments in response to the mechanical loading of the bursa-derived cells and the translocation of YAP into the nucleus. Cells were fixed in 4% PFA and permeabilized using 0.2% Triton (Sigma-Aldrich/Merck, Darmstadt, Germany) in tris-buffered solution (TBS, pH 8.2). Blocking was performed with 5% normal goat serum (NGS) and 1% bovine serum albumin (BSA) in TBS and subsequently the primary YAP antibody in Dako Antibody diluent (Dako North America, Carpinteria, CA, United States) was incubated at 4°C overnight. Goat-anti-mouse-Alexa Fluor 488 secondary antibody together with the Phalloidin-Alexa Fluor 647 Plus in blocking solution with 5% NGS and 1% BSA was added and incubated for 2 h at room temperature in the dark. Counterstaining was performed with DAPI and HCS CellMask Blue Stain (details for antibodies and probes see Table 1). In total, three representative fluorescence pictures were taken from each silicon dish per donor and loading condition with the BZ-X810 Keyence Epifluorescence microscope and the BZ-X800 software (Keyence, Osaka, Japan). The FibrilTool macro (Boudaoud et al., 2014) with a grid size of 75 pixels was used to analyze the orientation of the cells. The loading direction represents an orientation of 0° and a perpendicular orientation to the loading direction is given as ± 90°. The orientation and anisotropy of each grid were calculated by the macro. A very small anisotropy indicates no clear orientation in this grid for example in cases where no actin fibers or only small parts of a cell were visible in this respective grid. These orientation values were excluded. Histograms show cell orientation as a combination of all loadings. The average deviation from the mean orientation was calculated to assess the variances of orientations per loading condition and is given as fold to the unstimulated control. For the quantification of the cytosolic (cyt) and nuclear (nuc) YAP staining, a self-designed macro was used. The total cytosol area was set from actin staining and the nuclear area from DAPI staining. The YAP-positive area within the cytosolic area and nuclear area were calculated after using the Otsu method for automated thresholding. YAPcyt/YAPnuc ratios are given as fold to the unstimulated control. Representative images were additionally taken by employing a grid illumination sectioning to assess a z-stack. Maximum intensity projections were calculated using Imaris Cell Imaging Software (v9.6, Bitplane, Zurich, Switzerland).

**TABLE 1:**
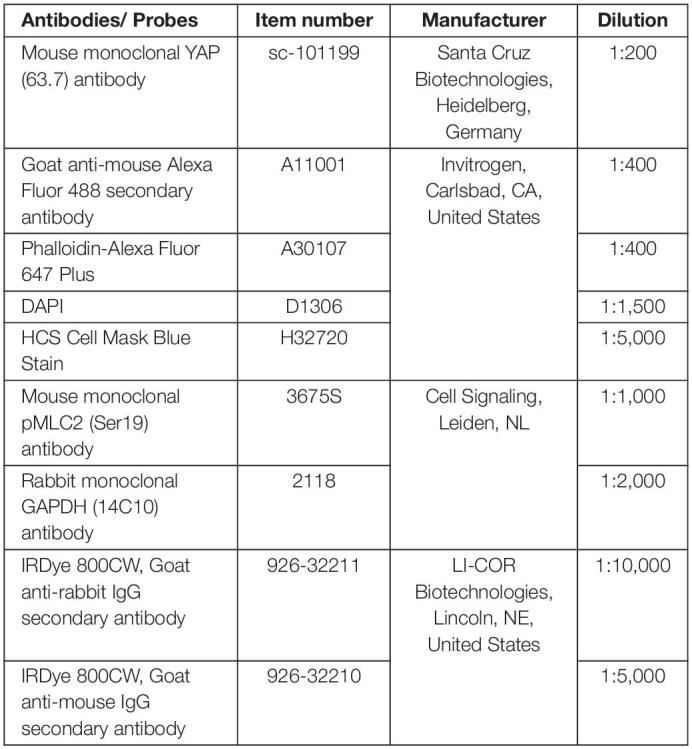
Antibodies and staining probes.

### Activation of NMM-II

Activation of NMM-II was evaluated by quantification of phosphorylated myosin light chain (pMLC). Cells were lysed directly in the silicon dishes using 1 × Laemmli buffer (62.5 mM Tris-HCl, pH 6.8, 10% glycerol, 2% SDS, 0.01% bromophenol blue, and 16.7 mM dithiothreitol) with protease inhibitor cocktail tablets (cOmplete Mini, Roche Diagnostics, Basel, Switzerland) on ice and afterward stored at –80°C. Protein samples were thawed and denatured at 85°C before loaded onto Novex^TM^ NuPage Bis-Tris Protein Gels together with the PageRuler Plus Prestained Protein Ladder (both Invitrogen, Carlsbad, CA, United States). Afterward, electrophoresis was run in 1 × Novex NuPage MES SDS running buffer (20×, Invitrogen) at 180 V until optimal protein separation. Blotting was performed at 30 V for 1 h. GAPDH and pMLC bands were separated and blocked with 3% BSA in 1 × TBS-Tween (BSA/TBS-T, 10×: Tris-HCl, NaCl, 1% Tween20, pH 7.6). The primary antibodies against pMLC2 in 5% milk powder/TBS-T solution with 0.5% sodium azide (NaN_3_) and GAPDH in 5% BSA/TBS-T solution with 0.5% NaN_3_ were incubated overnight at 4°C. The secondary antibodies were diluted in 3% BSA/TBS-T and incubated for 2 h at room temperature. Afterward, the membranes were imaged using the Odyssey imager (LI-COR Biotechnologies, Lincoln, NE, United States) with the Odyssey V3.0 software. The gray values of pMLC were analyzed and normalized to GAPDH and are given as fold values to the unstimulated control.

### Gene Expression Analysis

RNA was isolated from the bursa-derived cells directly in the silicon dish using the NucleoSpin RNA Kit (Machery-Nagel, Düren, Germany) according to the manufacturer’s manual. A total of 100 ng RNA was transcribed into cDNA with the qScript cDNA Supermix (Quanta Biosciences, Gaithersburg, MD, United States). Expression levels of integrins (ITGA1 and ITGA2), MMPs and TIMPs (MMP1, MMP2, MMP3, TIMP1, and TIMP2), and ECM components (COL1A1, COL3A1, Versican, and Fibromodulin) were measured using quantitative Real-Time PCR (QRT-PCR). QRT-PCR was performed with the SyBr Green Mastermix (Quanta Biosciences, Beverly, MA, United States) according to the manufacturer’s manual and the LightCycler 480 System (Roche, Mannheim, Germany). All primer sequences were designed using Primer 3 software (Freeware)^1^ and were produced by Tib Molbiol, Berlin, Germany (primer sequences see Table 2). All primers were tested for amplification efficiency, and the relative gene expression to the housekeeping gene Hypoxanthine phosphoribosyl transferase (HPRT) was calculated using an efficiency-corrected equation as described previously (Klatte-Schulz et al., 2018) and given as fold change to the control group. HPRT was tested to be the most constant housekeeping gene regarding the loading conditions compared to 60S ribosomal protein L13 (RPL13) and 18S rRNA.

**TABLE 2:** Primer for gene expression analysis in alphabetical order.

**Gene**	**Ascession number**	**Forward primer**	**Reverse primer**	**Size (bp)**
ACAN	NM_013227	CCAGTGCACAGAGGGGTTTG	TCCGAGGGTGCCGTGAG	146
COL1A1	NM_000088.3	TGACCTCAAGATGTGCCACT	ACCAGACATGCCTCTTGTCC	197
COL2A1	NM_033150.2	CGCACCTGCAGAGACCTGAA	TCTTCTTGGGAACGTTTGCTGG	162
COL3A1	NM_000090.3	AGCCTGGTAAGAATGGTGCC	TCCTTGCCATCTTCGCCTTT	103
DCN	NM _001920	CGCCTCATCTGAGGGAGCTT	TACTGGACCGGGTTGCTGAA	205
FMOD	NM_002023.4	CAACACCAACCTGGAGAACC	CAGCTTGGAGAAGTTCACGAC	103
HPRT	NM_000194	GAAGGTGAAGGT CGGAGTC	GAAGATGGTGAT GGGATTTC	211
ITGA1	NM_181501.1	TCAGGTGGGGATGGTAAGAC	TGGCTCAAAATTCATGGTCA	168
ITGA2	NM_002203.3	TCTCTTCGGATGGGAATGTT	CTGTTTGCACCCCAGTTAGG	196
MMP1	NM_002421.3	CACGCCAGATTTGCCAAGAG	GTCCCGATGATCTCCCCTGA	148
MMP2	NM_004530	TGGATGATGCCTTTGCTC GT	CCAGGAGTCCGTCCTTACCG	156
MMP3	NM_002422.3	TGGGCCAGGGATTAATGG AG	GGCCAATTTCATGAGCAGCA	104
S100A4	NM_002961.3	CCCTGGATGTGATGGTGTC	CACCTCGTTGTCCCTGTTG	188
TIMP1	NM_003254.2	TTGGCTGTGAGGAATGCA CA	AAGGTGACGGGACTGGAAGC	128
TIMP2	NM_003255.4	CCTGAGCACCACCCAGAAGA	TCCATCCAGAGGCACTCGTC	123
VCAN	NM_004385.4	GCTGCTCAGAAGGCTTGTTT	GACTCCTGCCTTTCCCATCT	183
VIM	NM_003380.5	CCTTGAACGCAAAGTGGAAT	GTGAGGTCAGGCTTGGAAAC	141

### Protein Secretion

Supernatants of bursa-derived cells were tested for Col I secretion using the MicroVue CICP EIA Kit (Quidel, San Diego, CA, United States) for determining levels of the C-Terminal propeptide of Type I Collagen (CICP). CICP values were calculated from the standard curve after measuring the optical density at 405 nm with the Tecan spectrophotometer. For quantification of the MMP and TIMP secretion under mechanical stimulation, the concentrations of total MMP1, MMP2, MMP3, and TIMP1 were determined using commercially available sandwich ELISAs (DuoSet ELISA, R&D Systems, Wiesbaden, Germany) according to the manufacturer’s manual. Col I, MMP, and TIMP concentrations were normalized to the total protein concentration measured using Pierce Coomassie Plus protein assay (Thermo Fisher Scientific, Waltham, MA, United States).

### Statistics

Statistical analysis was performed using GraphPad Prism version 7.0.0. for *n* = 4 to *n* = 6 individual donors per method as described in the experimental setup (Figure 2). The Kruskal–Wallis Test with Dunn’s Multiple Comparison was used to investigate significant differences between the three loading groups, the loading groups to the unstimulated control, as well as the 1 h stimulation groups with the 4 h stimulation groups. The level of significance was set at *p* ≤ 0.05. Additionally, a second *p*-value of *p* < 0.1 was used to indicate trends.

## Results

### Validation of Mechanical Strain Magnitudes in the Stretching Device

For the validation of the mechanical straining and its distribution across the whole cell culture dish, a speckle analysis was performed for five different strain magnitudes. Digital image correlation revealed that applying 2, 4, 8, 10, and 16% of stroke to the silicon dish holder resulted in a 35–38% reduced strain magnitude on the surface area of the silicon dish in tensional direction. This resulted in the planned strain magnitudes on the surface area. Perpendicular to the tensional direction, smaller compressive strains occurred. The minimal, maximal, and mean strain values are summarized in Table 3. For the sake of simplicity, in the following, the predominant tensional strain values were rounded up to whole numbers. The strain distribution on the surface area of the silicon dish is exemplarily depicted in Figure 1C for one stretched silicon dish at maximum position. The strain was lower at the corners of the fixed side of the silicon dish and higher in tensile direction (Figure 1C). The mean, minimal, and maximal strain values are shown in Table 3.

**TABLE 3:** Result of digital image correlation analysis (n = 4).

**Applied stroke**	**Tensional strain in tensional direction**				**Compressive strain perpendicular to tensional direction**		
	**Mean strain**	**Rounded strain**	**Mean min strain**	**Mean max strain**	**Mean strain**	**Mean min strain**	**Mean max strain**
2%	1.30 ± 0.2%	1%	0.83 ± 0.23%	1.57 ± 0.12%	0.90 ± 0.03%	0.57 ± 0.02%	1.08 ± 0.00%
4%	2.54 ± 0.3%	3%	1.72 ± 0.16%	3.05 ± 0.24%	1.63 ± 0.06%	1.13 ± 0.08%	2.06 ± 0.19%
8%	5.18 ± 0.3%	5%	3.79 ± 0.22%	6.29 ± 0.88%	3.08 ± 0.10%	2.17 ± 0.20%	3.67 ± 0.12%
10%	6.38 ± 0.3%	6%	4.72 ± 0.61%	7.45 ± 0.47%	3.74 ± 0.04%	2.99 ± 0.06%	4.15 ± 0.12%
16%	9.91 ± 0.5%	10%	7.44 ± 0.45%	11.97 ± 0.54%	5.58 ± 0.13%	3.94 ± 0.36%	6.42 ± 0.13%

### Characterization of Bursa-Derived Cells

As a precondition for the stimulation experiments, the isolated bursa-derived cells were characterized according to their expression profile of fibroblast-, stromal cell-, and ECM-related markers, which are expected to play a major role in bursa tissue. The bursa-derived cells expressed highest amounts of the fibroblast-associated marker Vimentin. The ECM marker COL1A1 and the proteoglycan Decorin were expressed in lower amounts similar to the stromal cell-associated marker S100A4. The expression of COL3A1, Aggrecan, and Versican showed comparable amounts but was lower compared to COL1A1 and Decorin. Lowest gene expression was observed for the proteoglycan Fibromodulin, whereas COL2A1 expression was not found in the cells (Supplementary Figure 1).

According to previous studies proving the progenitor cell potential of bursa-derived cells, the isolated cells were characterized for their surface marker expression, potential for self-renewal, and multipotent differentiation potential. Progenitor cell characteristics were clearly present in the bursa-derived cell cultures with ≥ 95% expression of the surface markers CD29, CD44, CD73, CD90, and CD105 and ≤ 2% expression of CD11b, CD14, CD34, and CD45. Cells of one donor showed lower levels of CD73 (85.8%) and CD90 (72.0%) and slightly elevated levels of CD11b (3.2%) and CD34 (9.3%). The results are presented together with comparative data from the literature in Supplementary Table 2. Regarding the colony-forming potential, an average of 17.4% of the bursa cells were able to form colonies. Additionally, bursa-derived cells showed the potential to differentiate into the osteogenic, adipogenic, and chondrogenic lineage as exemplarily depicted in Supplementary Figures 2A–C. Cell differentiation was confirmed on mRNA level (Supplementary Figure 2D). In summary, the investigated expression profile together with the analysis of the progenitor cell potential characterized the bursa-derived cells as fibroblastic cells with progenitor potential.

### Cell Morphology, Viability, and Orientation After Mechanical Loading

**FIGURE 3:**
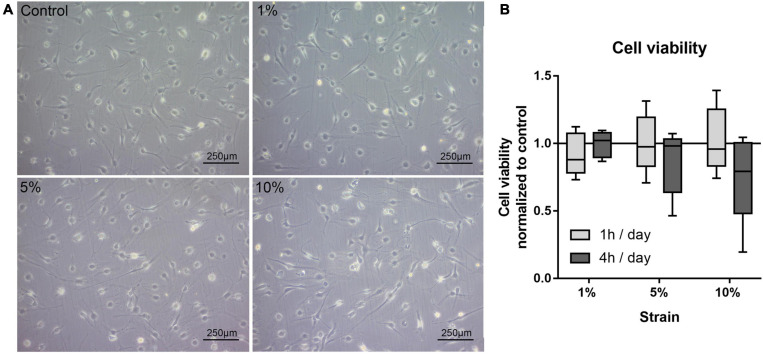
**(A)** Representative images of bursa-derived cell morphology taken from control cells and mechanically loaded cells from area b (middle) in the silicon dish. Cells were stimulated for 4 h per day at 1, 5, and 10% strain with 1 Hz. Scale bar: 250 μm. **(B)** Cell viability of bursa-derived cells after 1 and 4 h of mechanical loading per day. The values are given as fold to the unstimulated control. Statistics: Dunn’s multiple comparison test for *n* = 5 individual donors.

In this first setup, the reaction of bursa-derived cells to different loading conditions was tested. Morphological changes and cell viability, as well as possible adaptation in strain avoidance due to changes in cell orientation, were evaluated. The bursa-derived cells showed no distinct morphological changes after loading (Figure 3A). The cell viability remained stable for all 1 h loading conditions. For the 4 h stimulations, the cell viability tended to be reduced with 10% loading, but without statistical significance (Figure 3B).

**FIGURE 4:**
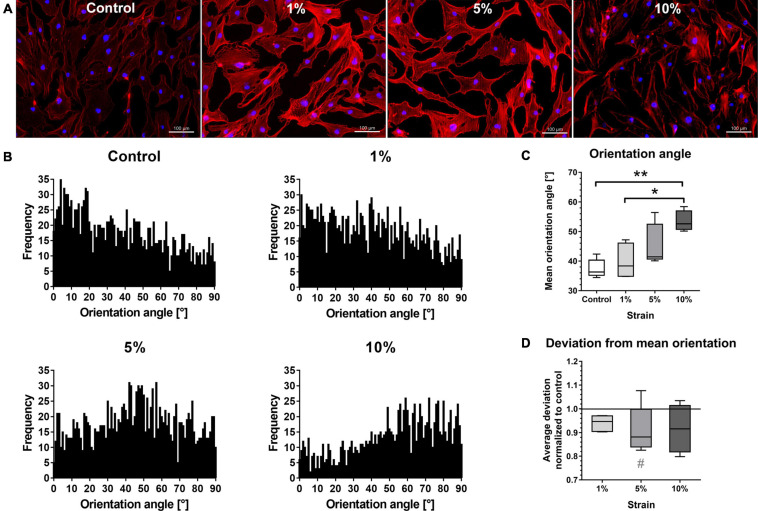
Morphology and orientation of bursa-derived cells stimulated at 3 days for 4 h per day at 1 Hz with 1, 5, and 10% strain compared to the unstimulated control. **(A)** Representative z-stack images of Phalloidin and DAPI staining of actin filaments for all loading and control conditions. Representative images derived from area b (middle) in the silicon dish. Left to right = tensional direction. Scale bar: 100 μm. **(B)** Total frequencies of the orientation angles measured using the FibrilTool macro from images taken at three positions (a, b, c) in the silicon dish from five individual donors (0° = loading direction; 90° = perpendicular to the loading direction). **(C)** Mean orientation angles of all loading conditions calculated from the mean orientation of each individual image and given as absolute degree values. **(D)** Average deviation from mean orientation angle calculated from the mean values of each individual image and given as fold to the unstimulated control (line at 1). Statistics: Dunn’s multiple comparison test for *n* = 5 individual donors. A spanning line with asterix marks significant differences with a *p*-value of <0.05 and a spanning line with two asterix marks significant difference with a *p*-value of <0.01. The gray # indicates trend significant differences with a *p*-value of < 0.1.

The bursa-derived cells of the 1 and 5% loading group had a denser actin cytoskeleton with distinct actin fibers compared to the unstimulated control, whereas with 10% loading, the cells looked more stressed with loss in actin density and a partially destroyed actin cytoskeleton (Figure 4A). Although the orientation angels showed a high variability, a shift from the loading angles in the control and 1% loading to higher angels in the 5% and 10% stimulation group was visible in the histograms (Figure 4B). When investigating the mean orientation angles calculated from the mean orientation of each image, the cells seem to orient in the direction in-between the maximum tensional (0°) and maximum compressive (90°) strains (Figure 4C). The highest mean orientation angle of 53.4° was observed for the 10% loading group, which was significantly different compared to the control (37.5°, *p* = 0.008) and the 1% loading group (40.0°, *p* = 0.040) (Figure 4C). Despite the variation of orientation angles, the most uniform cellular orientation was observed for the 5% loading group with, by trend, the lowest average orientation from the mean orientation (*p* = 0.094, Figure 4D). Altogether, these data suggest a direct response of the bursa-derived cells to different loading conditions by adaptation of cell orientation and hint for induction of pathological conditions in the 10% loading group.

### Analysis of Mechano-Transduction Pathways

**FIGURE 5:**
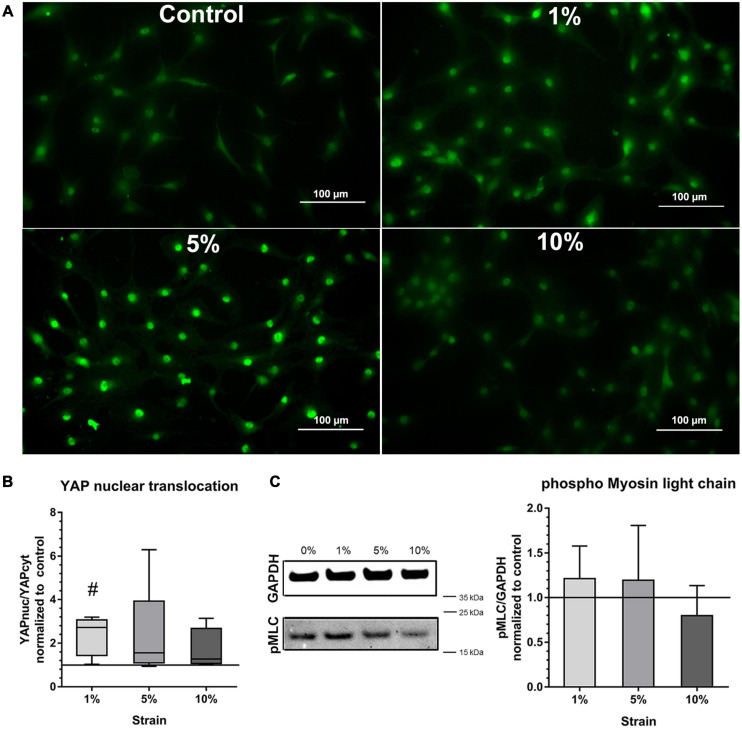
Mechano-transduction pathways in bursa-derived cells stimulated at 3 days for 4 h per day at 1 Hz with 1, 5, and 10% strain compared to the unstimulated control. **(A)** Exemplary images of YAP fluorescence staining for all loading and control conditions. These exemplary images resulted from bursa-derived cells with highest Yap nuclear translocation in the 1 and 5% group in the study. Exemplary images derived from area b (middle) in the silicon dish. Left to right = tensional direction. Scale: 100 μm. **(B)** YAP nuclear translocation calculated as the ratio between the YAP-positive nucleus area and the YAP-positive cytoplasm area and given as fold to the unstimulated control (line at 1). Statistics: Dunn’s multiple comparison test for *n* = 5 individual donors. The # indicates significant differences with a *p* < 0.05. **(C)** Representative images of pMLC bands and GAPDH reference bands from Western blot analysis. Images are cropped and original images are provided in Supplementary Materials. Images were taken at a wavelength of 800 nm using the Odyssey V3.0 software with a resolution of 42 μm and an intensity of 4 for GAPDH and 8 for pMLC. The bar graphs show pMLC quantification relative to GAPDH and given as fold to the unstimulated control (line at 1). Statistics: Dunn’s multiple comparison test for *n* = 4 (*n* = 3 for 5% group) individual donors.

To prove further the mechano-responsiveness of bursa-derived cells to mechanical loading, mechano-transduction pathways were investigated. YAP activation, indicated by the presence of YAP in the nucleus, was determined in all stimulation conditions including the unstimulated control. Stronger nuclear YAP staining was observed for the 1 and 5% loading group (Figure 5A). A significant increase in YAP activation as shown by a higher YAPnuc/YAPcyt ratio could be observed in the 1% loading group compared to the unstimulated control (*p* = 0.012). A similar trend was present for the 5% loading group, but the high standard deviation prevented statistical significance (Figure 5B).

Activation of NMM-II through phosphorylation of MLC measured by Western blot analysis showed a similar trend compared to the YAP activation with a slight increase in the 1 and 5% loading group, but the signal was very weak and no significant differences occurred (Figure 5C). This analysis revealed partial activation of mechano-transduction pathways in the bursa-derived cells, proving their mechano-responsiveness especially for the 1 and 5% loading condition.

### Gene Expression Analysis

**FIGURE 6:**
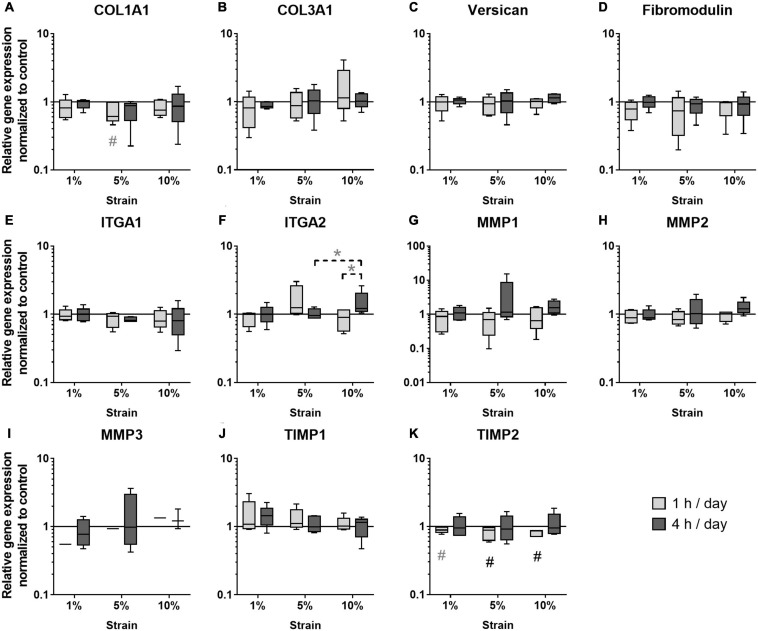
Gene expression of ECM markers **(A–D)**, integrins **(E,F)**, MMPs **(G–I)**, and TIMPs **(J,K)** in bursa-derived cells after 1 h and 4 h of mechanical loading per day for 3 days at 1 Hz. Values are given as normalized expression to the housekeeping gene HPRT and the unstimulated control (line at 1) using an efficiency corrected formula. Statistics: Dunn’s multiple comparison test for *n* = 5 individual donors. The # marks significant differences to the unstimulated control group with a *p* < 0.05. A dashed spanning line with gray asterix or a gray # marks differences with a *p*-value of < 0.1.

The next step was to study the effect of different loading conditions on the expression of genes important for ECM formation and remodeling. In the group of the ECM markers, COL1A1 expression tended to be decreased with 5% loading at the 1 h stimulation time (*p* = 0.071, Figure 6A), whereas COL3A1 expression was not regulated by mechanical loading (Figure 6B). The expression of Versican and Fibromodulin was unchanged in mechanically stimulated bursa-derived cells (Figures 6C,D). ITGA1 expression was not strain-dependently regulated and ITGA2 expression strain-dependently increased by trend in the 10% loading group for 4 h compared to the 5% group (*p* = 0.094) and the respective 1 h loading time (*p* = 0.094, Figures 6E,F). Basal expression levels of ITGA1 were higher compared to ITGA2. The expression of the collagenase MMP1 and the gelatinase MMP2 was not regulated by the applied loading conditions in the bursa-derived cells (Figures 6G,H). MMP3 expression was in general low and only measurable in one of the five donors in the 1 h stimulations, whereas after 4 h of loading, MMP3 expression appeared in four of the five donors without significant difference between the loading groups (Figure 6I). Basal MMP2 expression levels were highest within all measured MMPs. TIMP1 expression levels were high and unchanged in the bursa-derived cells under the tested conditions (Figure 6J). For TIMP2, a significantly reduced expression was observed for the 5 and 10% loading group for 1 h stimulations compared to the unstimulated control (*p* = 0.021 and *p* = 0.005, respectively). Additionally, a trend toward a decreased TIMP2 expression was also seen for the 1% loading group (*p* = 0.082, Figure 6K). Interestingly, the analysis on the gene expression level revealed only few significant differences between the groups. The decrease in TIMP2 expression hints for an MMP/TIMP imbalance at higher loading conditions.

### Protein Secretion

**FIGURE 7:**
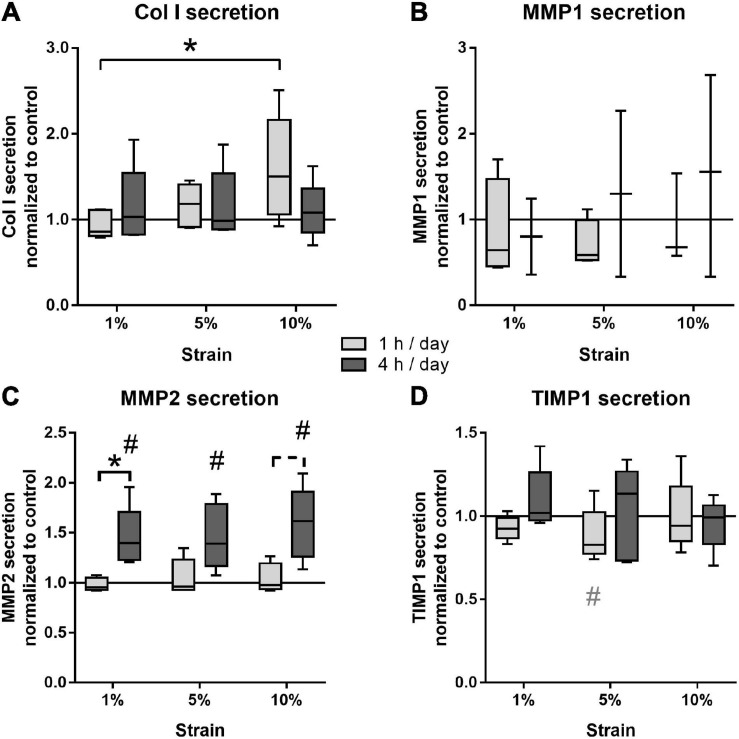
Protein secretion of Col I **(A)**, MMP1 **(B)**, MMP2 **(C)**, and TIMP1 **(D)** from bursa-derived cells after 1 and 4 h of mechanical loading per day for 3 days at 1 Hz. The values are given as fold to the unstimulated control cells (line at 1). MMP1 secretion was below the detection limit in one or two of five donors for the 1 and 4 h stimulations, respectively. MMP3 secretion was below the detection limit in all samples. Statistics: Dunn’s multiple comparison test for *n* = 5 individual donors. A spanning line with asterisk or a # marks significant difference with a *p*-value of < 0.05. A dashed spanning line or a gray # marks differences with a *p*-value of < 0.1.

The effect of different loading conditions on bursa-derived cells was finally investigated on the protein level. Col I secretion was significantly increased in the bursa-derived cells after 1 h of 10% loading compared to the cells loaded at 1% strain (*p* = 0.027). Loading for 4 h per day resulted in an equal Col I secretion for all strain magnitudes compared to the unstimulated cells (Figure 7A). MMP1 secretion was very low and partially below the detection limit of the assay. In the 1 h stimulation groups, no significant regulations were observed, whereas a slight strain-dependently increased was found in the 4 h stimulation groups, which did not reach significant differences due to the limited sample size (Figure 7B). The secretion of MMP2 to the cell culture supernatant showed highest protein levels of all analyzed MMPs. The 1 h stimulations resulted in similar MMP2 secretion between all stimulation conditions. In contrast, in the 4 h stimulations, the MMP2 secretion was significantly increased for all loading conditions compared to the control group (*p* = 0.029, *p* = 0.034, and *p* = 0.009) and the respective 1 h stimulations in the 1% (*p* = 0.028) and, by trend, the 10% loading group (*p* = 0.058, Figure 7C). MMP3 secretion was below the detection limit of the assay in all samples. TIMP1 protein secretion was not significantly regulated by mechanical loading, but a trend toward a decreased TIMP1 secretion was observed for the 1 h stimulations with 5% loading compared to the unloaded control (*p* = 0.071; Figure 7D). In summary, the analysis on the protein level clearly indicates that bursa-derived cells adapt their ECM secretion and remodeling processes especially in response to intense and longer mechanical stimuli.

## Discussion

While it is well known that cells of directly mechanically loaded tissues show a clear mechano-responsiveness, this was so far not known for adjacent indirectly loaded tissues such as the subacromial bursa. To better understand the physiological function of the subacromial bursa as a friction-reducing structure in the shoulder, it was essential for us to understand whether and how bursa-derived cells are mechano-responsive and react to physiological and pathological mechanical loading regimes. Therefore, mechano-transduction pathways such as YAP nuclear translocation and NMM-II activation as well as the ability of the cells for adapting the ECM formation and remodeling were evaluated. The overall results of the present study show that bursa-derived cells respond to mechanical signals and show specific reactions to distinct strain magnitudes. As a basis for future studies in this field, this knowledge might lead to optimized surgical and/or physiotherapeutic treatment strategies in the future.

We used a mechanical stretching device, adapted to a device first published by Neidlinger-Wilke et al. (1994). The main difference is that smaller silicon dishes with 2 × 3 cm surface area were used compared to 3 × 6 cm surface area, which reduced the needed number of human primary cells. The validation of the strain magnitudes on the silicon surfaces was performed using a speckle analysis and digital image correlation (Bieler et al., 2009; Seefried et al., 2010). Digital image correlation analysis was rated as robust optical method for measuring tension (Zhang and Arola, 2004). Lower strain values occurred at the corners of the surface area and higher values occurred at the center of the surface area in stretching direction. Perpendicular to the tensional direction, compressive strains occurred to a lesser extent. For the predominant tensional strain, variations from minimal to maximal strain values are comparable to values reported in the literature (Seefried et al., 2010). Independent of local variations in strain magnitude within a single surface area, external loads leading to low, medium, or high strain values did not overlap in their strain ranges and thus illustrate ranges of physiological or pathological straining conditions. Also in *in vivo* situations, it may be expected that different locations of the subacromial bursa would undergo different strain magnitudes. Such an inhomogeneous straining of the bursa might be similar to settings in directly loaded neighboring tissues such as the supraspinatus tendon, where strain values vary depending on the localization and adjacent tissues during locomotion (Huang et al., 2005).

The presently used bursa-derived cells showed a fibroblast-associated expression profile with highest amounts of Vimentin gene expression and lower expression of S100A4, a stromal cell-associated marker. Regarding the expression of ECM genes, the bursa-derived cells showed similar expression of COL1A1 and the proteoglycan Decorin. Aggrecan expression, a proteoglycan important for resistance against compressive loads (Yoon and Halper, 2005), as well as COL3A1 and Versican expression were lower and fibromodulin expression was lowest compared to all other tested ECM markers. The analysis of the surface marker phenotype, the ability for self-renewal, and the multipotent differentiation potential proved the progenitor cell potential of the bursa-derived cells. This also confirms other studies showing that bursa cells have a stromal cell potential (Steinert et al., 2015; Morikawa et al., 2019). No specific markers for bursa-derived cells are known to clearly identify them. The investigated expression profile together with the analysis of the progenitor cell potential characterized them as fibroblastic cells with progenitor potential as also described by others (Utsunomiya et al., 2013; Steinert et al., 2015; Morikawa et al., 2019).

As little is known about mechanical straining of the subacromial bursa *in vivo*, physiological and pathological strain values were defined according to values measured from directly loaded tissues such as the neighboring tendons. It was shown for tendons that up to 5% of strain represents physiological conditions, whereas 8% strain or higher leads to tissue rupture (Arnoczky et al., 2007). The present study utilized a 2D *in vitro* culture model, which is limited in optimally mimicking the *in vivo* situation. For loading of bursa tissue *in vivo*, compression might be more relevant than tension. However, despite the predominance of tensional strain in the present loading setup, compressive strains occurred perpendicular to the tensional direction. Additionally, it is known *in vivo* that compression will result in tension forces in other regions of tissues (Gracey et al., 2020). Therefore, compressive and tensional strain cannot be viewed independent from each other. Regardless of the type of stimulation, this pilot study aimed at investigating if bursa-derived cells are able to sense and respond to mechanical signals. This is expected to be independent from the actual stimulation conditions. The cell viability remained stable between all analyzed strain magnitudes for the 1 h stimulation time, but a trend toward a reduced cell viability with 10% strain was observed for 4 h stimulations, indicating a stress response of the cells according to overloading conditions. This result goes along with the loss in actin density and induced damage of the actin cytoskeleton in the 10% loading group and the missing activation of the mechano-transduction pathways in this loading condition. Regarding cell viability/proliferation, others reported an increase in cell proliferation of osteoblasts stimulated with low strain values of 1% for 15 min per day, whereas higher strain magnitudes had no effect (Neidlinger-Wilke et al., 1994). In contrast, high strains of 10% for 8 h per day for 5 days led to an increase in cell proliferation of mesenchymal stem cells (MSCs) and anterior cruciate ligament fibroblasts (Sun et al., 2016). In general, a wide range of different loading regimes are used for different cell types, which makes direct comparison difficult. The data demonstrate that 10% loading for 4 h over 3 days induces an overloading condition in the bursa-derived cells. Further analysis has to be conducted to investigate the consequence of this overloading for the bursa-derived cells such as apoptosis or induction of pro-inflammatory signaling cascades.

To prove the responsiveness of the bursa-derived cells to mechanical stimulation, adaptation of cell orientation and the activation of mechano-transduction pathways were investigated, which are indicators for the ability of the cells to sense mechanical signals. When investigating the orientation of the cytoskeleton of the bursa-derived cells, the orientation angles showed a high variability in combination of the five individual donors. However, a shift from the angles in the control and 1% stimulation group to a direction in-between the maximum tensional (0°) and maximum compressive (90°) strains in the 5% as well as 10% stimulation group was visible. In doing so, the cells react to the mechanical stimuli and appear to prevent high strain magnitudes, by preferably orienting in the direction with reduced deformity of the silicon surface, known as “strain avoidance”. The high variability in orientation angles might partially be explained by the morphology of the bursa-derived cells, which have several focal adhesion points not only orienting in one direction. Additionally, different constellations of compressive and tensional strains on the silicon surface might have avoided full uniform orientation. This result of strain avoidance supports other findings of human MSCs, which strain- and time-dependently oriented perpendicular to the stretching direction particularly when loaded for 24 and 48 h (Morita et al., 2013; Nam et al., 2019). Additionally, the bursa-derived cells showed a denser actin cytoskeleton after 4 h of loading, which is in accordance with findings of MSCs (Nam et al., 2019). YAP activation was significantly increased in the 1% loading group with a similar trend for the 5% loading group compared to the control. In general, YAP activation with a YAP nuclear translocation was present for all stimulation conditions and the unstimulated control. This is due to the limitations of the method, as accompanying factors such as ECM stiffness, cell shape, and cell confluence are also affecting YAP activity (Dupont et al., 2011). A stiff ECM, such as the present silicon dishes, thereby leads already to YAP nuclear translocation, which makes the detection of differences in YAP activation more difficult. However, significant translocation occurred in the present study, proving an additional YAP activation by mechanical loading. Similarly, NMM-II activation is also dependent on the stiffness of the matrix (Raab et al., 2012; Shin et al., 2013). Additionally, NMM-II levels in general are highly variable between different cell types and differentiation status (Shin et al., 2013). Especially in non-muscle cells with low myosin II content, large numbers of cells are needed to detect sufficient amounts of pMLC (Kazakova et al., 2019). This might explain the weak pMLC signal, resulting in minor differences between the loading conditions. Overall, the cellular orientation and increased density of the actin cytoskeleton as well as the significant up-regulation of the YAP activation indicates a clear mechano-responsiveness of the bursa-derived cells.

Physiological or pathological tissue straining may alter gene expressions, which might be close to those found in other connective tissues (Wang et al., 2007). Therefore, to investigate how subacromial bursa-derived cells respond to mechanical stimulation, selected genes related to the ECM formation and remodeling as well as respective protein secretion were analyzed. Integrins are transmembrane receptors that connect the actin cytoskeleton to the ECM. By this, mechanical stimuli are transmitted to the inner of the cell and the nucleus. ITGA1 and ITGA2 are collagen-binding integrins and showed slightly different mechano-dependent alterations in the present study. ITGA1 expression was unchanged, whereas ITGA2 showed a trend toward an increased gene expression with higher loading. This is partially in accordance with other findings showing that human tendon stem cells stimulated with the same uniaxial system, as presently used, up-regulated their ITGA1 and ITGA2 expression after 1 h of stimulation for 3 days (Popov et al., 2015). Furthermore, a study with human MSCs mechanically stretched at 1% strain on nanofiber scaffolds reported an increased ITGA2 expression starting after 7 days of stimulation or later (Subramony et al., 2013). In a previous study with mouse tenocytes stimulated with the equiaxial flex cell system, ITGA1 was also unchanged but ITGA2 expression decreased after 4% and 4 Hz loading for 5 days (Fleischhacker et al., 2020). It seems obvious that regulation of Integrin expression is highly dependent on the substrate, cell type, and time of stimulation. An up-regulation of integrin expression was described as necessary to provide the sufficient number of transmembrane receptors to sense mechanical stimulation (Subramony et al., 2013). It seems that ITGA2 up-regulation is more important for bursa-derived cells to sense mechanical signals. However, regulation of integrins was not strongly pronounced under the tested conditions, indicating a probably sufficient amount of integrins or hinting for non-optimal timing of ITGA gene expression analysis.

Mechanical loading of tissues *in vivo*, either compression of bone or tension of tendons, was mostly described to have anabolic effects on ECM production (Heinemeier et al., 2007; Holguin et al., 2013; Zhang and Wang, 2013; Birkhold et al., 2017). For the subacromial bursa, it is known that chronic degenerative processes that are probably caused by overloading stresses induce changes in tissue structure regarding increased matrix and villi formation, synovial hyperplasia, and increased cellularity (Ishii et al., 1997; Benson et al., 2009; Chillemi et al., 2016). Therefore, it could be expected, that *in vitro* loading resulted in similar adaptations in ECM formation. Presently, *in vitro* loading of bursa-derived cells led to the expected increase in Col I protein secretion for short time stimulation, as also reported by others for MSCs and tenocytes (Zhang et al., 2008; Huisman et al., 2014). No up-regulation of Col I protein secretion was observed for 4 h stimulations, which might be explained by the increased stress for the cells leading to discontinuation of matrix production. In contrast, on the gene expression level, a trend toward a decreased COL1A1 expression at short-term stimulation occurred, whereas COL3A1 expression was not influenced by mechanical loading. When reviewing the literature, it seems that, among others, the duration of stimulation is affecting the results. Shorter stimulation periods of 1–3 h led to no changes in collagen gene expression in tendon cells (Popov et al., 2015; Fleischhacker et al., 2020), whereas longer stimulation periods of 12–48 h resulted in increased collagen gene expression in MSCs (Zhang et al., 2008; Morita et al., 2013). On the other hand, the regulation might also be dependent on the cell type and substrate the cells are seeded on, as tenocytes grown on Col I coated surfaces and stimulated for 12 h at 4% strain also showed a decreased Col I gene expression (Joo Kim et al., 2016). Versican and Fibromodulin expression was presently not regulated by the applied loading conditions, which is in contrast to findings of increased expression in tendon stem cells stimulated with the same stretching device, but different surface coating (Popov et al., 2015). It seems obvious that the different loading regimes and cell culture conditions have a major impact on the mechano-regulation of ECM gene expression, which makes direct comparisons difficult. The presently applied loading conditions did not elicit anabolic reactions on the gene expression level in bursa-derived cells, whereas Col I protein secretion was significantly increased. This difference might be explained by posttranslational modifications that regulate collagen synthesis. Furthermore, gene expression analysis only depicts a short period of stimulation and might be highly dependent on the timing of RNA isolation, whereas the Col I protein was enriched in the cell culture medium and displays the entire stimulation period of 3 days. Regardless of the higher variance in cell orientation, the bursa-derived cells seem to partially undergo strain avoidance by orienting in a direction in-between maximum tensional and compressive strain over time with increased loading. In these cases, strain magnitudes in the bursa-derived cells would change and might additionally affect results on the gene expression level.

MMPs and their natural inhibitors, the TIMPs, play an important role in tissue remodeling and their balance is crucial to maintain tissue homeostasis. With this, they indirectly contribute to the adaptation of ECM composition. MMPs are downstream targets of integrins and their mechano-regulation is reported for different cell types (Fujisawa et al., 1999; Yang et al., 2005; Sasaki et al., 2007; Popov et al., 2015). Presently, MMP gene expression was not regulated under the tested conditions, which can be explained by their posttranslational regulation mechanisms. In contrast, TIMP2 expression was decreased strain-dependently in long time stimulations. Previously, we found no significant regulations of MMP2, MMP3, TIMP1, and TIMP2 expression in mouse tenocytes stimulate with the flexcell system at 4% strain on collagen coatings (Fleischhacker et al., 2020). No mechano-regulation of MMP1, MMP2, and MMP3 was also reported for tendon stem cells seeded on FCS-coated dishes and stimulated for 1 or 3 days at 1, 5, and 8% strain (Popov et al., 2015). In contrast, MMP2 and TIMP2 expression increased in tendon cells stimulated at 10% strain using the flexcell system with collagen coating (Huisman et al., 2016). Altogether, the regulations on the gene expression level displays an MMP-TIMP imbalance with a shift in the direction of a higher MMP presence with higher/longer straining. This was underlined on a protein level with an additional increase in MMP2 secretion and a decrease in TIMP1 secretion. ECM remodeling is in general a continuous physiological response of connective tissues to mechanical loading. However, high strains can also induce tissue damage *in vivo* (Arnoczky et al., 2007) and the bursa-derived cells seem to adapt to these strains by up-regulating remodeling processes to compensate for this effect especially in long time stimulations. As MMPs are downstream targets of inflammatory cytokines (Xie et al., 2004; Sundararaj et al., 2009), their shift toward an increased MMP production might be caused by the induction of inflammatory pathways due to the overloading conditions.

Some limitations have to be mentioned for the present study. First of all, it might be assumed that for the subacromial bursa as a buffering system, compression forces are *in vivo* more relevant than stretching. In the present *in vitro* setup, compression strains occurred, to a lesser extent, perpendicular to the tensional direction. Furthermore, these forces cannot be viewed independent from each other, as compression will result in tension forces in other regions of the tissue (Gracey et al., 2020). Furthermore, the analysis was only performed in a 2D environment and a direct transfer to the *in vivo* situation is critical. With this pilot study, we aimed at understanding if bursa-derived cells are able to respond to different mechanical loading regimes instead of mimicking an optimal *in vivo* situation. However, with the mechanical stretching device, 3D investigations might be possible in the future. Another limitation is that regulations on transcriptional level are highly dependent on time-related changes. The analysis on RNA level is limited to one time point, which was 2 h after stimulation, when the RNA was isolated. Some regulations on the gene expression level might therefore not be recognized due to wrong timing. Time kinetic changes will be investigated in future studies. The subacromial bursa was hypothesized for being able to augment rotator cuff healing, but the bursa-derived cells used for the present study were obtained from a different degenerative shoulder pathology (omarthrosis and necrosis of the humeral head). It can only be speculated if bursa-derived cells from patients with rotator cuff lesion or healthy patients have the same mechanical competence.

## Conclusion

In summary, the findings of the present study show a clear mechano-responsiveness of bursa-derived cells illustrated by their cytoskeletal organization, their YAP activation, as well as their increase in ECM formation and remodeling. Beyond a “physiological strain” limit, a long time stimulation with 10% strain seems to induce an overloading condition in these cells. This also indicates that bursa tissues and their embedded cells are highly sensitive to specific mechanical strain values. When looking for regulations under straining that are associated with adaptation of ECM formation and remodeling, it becomes clear that higher strain magnitudes led to increased Col I secretion and an MMP/TIMP imbalance toward increased MMP activity. These findings indicate that bursa-derived cells are reactive to high mechanical tissue straining by adapting their matrix turnover and enhancing remodeling. This pilot study provides first insights into the mechano-responsiveness of bursa-derived cells, a tissue that is only considered to be indirectly mechanically loaded. Our data indicate that the view on the role of bursa tissue may have to be changed and that it is relevant to understand in more detail the mechano-responsiveness in physiology and pathology to gain a more comprehensive knowledge on the role of the bursa in the shoulder joint. With the data shown here, the study provides the basis for further investigations that might lead to optimized physiotherapeutic treatments or surgical procedures in the future.

## Data Availability Statement

The original contributions presented in the study are included in the article/Supplementary Material, further inquiries can be directed to the corresponding author/s.

## Ethics Statement

The studies involving human participants were reviewed and approved by the Ethics Commission, Charité-Universitätsmedzin Berlin. The patients/participants provided their written informed consent to participate in this study.

## Author Contributions

FK-S and BW: conceptualization and funding acquisition. IV, JM, AS, NB, and CB: investigation and methodology. FK-S, IV, and CB: visualization. KT, PM, MH-L, and AI: resources. FK-S, GD, and BW: supervision. FK-S: writing—original draft. MH-L, AI, CB, GD, and BW: writing—review and editing. All authors have read and agreed to the published version of the manuscript.

## Conflict of Interest

The authors declare that the research was conducted in the absence of any commercial or financial relationships that could be construed as a potential conflict of interest.

## Footnotes

1 http://frodo.wi.mit.edu/primer3
