# Supplementary material for: Bursa-Derived Cells Show a Distinct Mechano-Response to Physiological and Pathological Loading in vitro
Source: Front Cell Dev Biol. 2021 May 31;9:657166. doi: 10.3389/fcell.2021.657166 (PMC8201779; doi:10.3389/fcell.2021.657166)
Supplement: Supplementary file 1 [file Data_Sheet_1.pdf]

Supplementary Material

1 Supplementary Data

1.1 Material and methods for investigation of progenitor cell potential:

Exemplary analysis of the progenitor cell potential was performed from 3 bursa-derived cell cultures and were analyzed for the fulfillment of the minimal stromal cell criteria. For investigation of progenitor cell phenotype, bursa cells at passage 1 were stained for the positive stem cell markers CD29 (PE, 1:500), CD44 (PECy7, 1:5000), CD73 (APC, 1:1000), CD90 (PerCPCy5.5, 1:400) and CD105 (FITC, 1:50)(all Biolegend), the negative markers CD11b (V450, 1:50), CD14 (APCCy7, 1:50), CD19 (PE, 1:50), CD45 (PerCP, 1:50)(all BD Biosciences) and CD34 (FITC, Biolegend, 1:50) as well as with Live/Dead reagent (1:500; Invitrogen). Unstained cells served as control. After staining, cells were washed with FACS buffer (PBS + 1% FCS) and fixed with 1% PFA. Measurements were performed at the BD FACSCanto II system (BD Biosciences) and data analysis with the FlowJo 9.6.4 software. To analyze the potential for self-renewal, a colony forming unit assay (CFU-Assay) was performed. A total of 1000 vital cells were cultivated for 10 days in cell culture medium and after fixation with 4% PFA colonies were stained with 1% methylene blue in borate buffer / 1% azur in dH2O (1:1, Sigma-Aldrich) for 10 min. Pictures were taken and the amount of colonies was counted via ImageJ 1.48v. For investigating the multipotent differentiation potential, the isolated bursa-derived cells were stimulated in passage 2-3 with osteogenic media (DMEM low glucose, 10% FCS, 1% P/S, 500 µM L-Ascorbic acid 2 phosphate, 10 mM β-Glycerolphosphate and 100 nM Dexamethasone) for 2 weeks, and adipogenic media (1 µM Dexamethasone, 2 µM Insulin, 0,5 mM Isobutylmethylxanthine und 60 µM Indomethacine) or normal growth medium for 3 weeks with 2 medium changes per week. All cell culture supplements were obtained from Sigma-Aldrich. Osteogenic matrix formation was visualized by 0.5% Alizarin Red S staining (Sigma-Aldrich) in 0.5 M HCl for 10 min. Furthermore, Alkaline phosphatase (AP) was stained with an AP staining solution (0.06% Fast Blue BB Salt (Waldeck, Muenster, Germany), 0.01% Naphtol-AS-MX-Phosphate, 0.5% dimethylformamide (Sigma-Aldrich), 2 mM magnesium chloride (Merck), and 0.1 M Trisbase in dH2O, pH 8.5) for 30 min at 37°C. To stain the lipid-vacuoles in the adipogenic differentiated cells, 0.3% Oil Red O (Sigma-Aldrich) in 99% isopropanol was applied for 10 min. For chondrogenic differentiation, $2.6\text{-}3*10^5$ vital cells were pelleted in 15 ml Tubes and chondrogenic media was added: DMEM high glucose, 1% P/S, 100 nM Dexamethasone, 175 µM L-Ascorbic acid 2-phosphate, 0.35 mM L-Proline, 6.25 µg/ml Insulin-transferrin-sodium-selenite-supplement, 1.25 mg/ml bovine serum albumin, 19.1 µM Linolenic acid und 10 ng/ml TGF-β1 (all Sigma Aldrich), and 2 mM Sodium pyruvate (Biochrom AG). After 5 weeks, cell pellets were fixed with 4% PFA and embedded into paraffin. Slices 4 µm thick were prepared and stained with H&E staining, 1% Alcian blue (Sigma-Aldrich) and Collagen type II (monoclonal mouse anti-human Collagen type II, clone 2B1.5; 1:25, Quartett, Berlin, Germany). One sample of the triplicates was analyzed on mRNA level. To grind chondrogenic cell pellets, a Minilys homogenisator (Precellys, Peqlab) with Trifast peqGOLD (Peqlab) and ceramic beats (Ø 1.4 mm) was used. Chloroform was added and after centrifugation, the aqueous phase was enriched with isopropanol and glycogen (Roche Diagnostics) to extract the RNA. Afterwards RNA was purified using the NucleoSpin RNA Kit. QRT-PCR analysis was performed as described in the qRT-PCR section for the differentiation markers relative to the housekeeping gene 18s rRNA as seen in supplementary table 1.

1.2 Supplementary Table:

Supplementary Table 1: Primer for differentiation experiments

Gene	Accession No.	Primer sequence
18S rRNA	**NM_022551**	Forward: 5' CGGAAAATAGCCTTTGCCATC 3'<br>Reverse: 5' AGTTCTCCCGCCCTCTTGGT 3'
FABP4	**NM_001442**	Forward: 5' ACTGGGCCAGGAATTTGACG 3'<br>Reverse: 5' ATGACGCATTCCACCACCAG 3'
PPARγ	**NM_015869**	Forward: 5' AAAGTCCTTCCCGCTGACCA 3'<br>Reverse: 5' GGCCACCTCTTTGCTCTGCT 3'
LEP	**NM_000230**	Forward: 5' TGCGGATTCTTGTGGCTTTG 3'<br>Reverse: 5' AAGGTCAGGATGGGGTGGAG 3'
ACAN	**NM_013227**	Forward: 5' CCAGTGCACAGAGGGGTTTG 3'<br>Reverse: 5' TCCGAGGGTGCCGTGAG 3'
COL2A1	**NM_033150.2**	Forward: 5' CGCACCTGCAGAGACCTGAA 3'<br>Reverse: 5' TCTTCTTGGGAACGTTTGCTGG 3'
COMP	**NM_000095.3**	Forward: 5' GCAACACGGACGAGGACAAG 3'<br>Reverse: 5' CGCCATCACTGTCCTTCTGG 3'
AP	**NM_000478**	Forward: 5' GGAAATCTGTGGGCATTGTG 3'<br>Reverse: 5' CCCTGATGTTATGCATGAGC 3'
OC (BGLAP)	**NM_199173.4**	Forward: 5' CCCAGGCGCTACCTGTATCAA 3'<br>Reverse: 5' CTGGAGAGGAGCAGAACTGG 3'
RUNX2	**NM_103532**	Forward: 5' GCCCCCAAACAGTATCTTGA 3'<br>Reverse: 5' GCCTGAAGTGAGGTTTTAGGC 3'

FABP4 = fatty acid binding protein 4, *PPARγ* = peroxisome proliferator-activated receptors γ, *LEP* = leptin, *ACAN* = aggrecan, *COL2A1* = collagen type II A1, *COMP* = cartilage oligomeric matrix protein, *AP* = alkaline phosphatase, *OC* = osteocalcin, *RUNX2* = runt-related transcription factor 2
