# Supplementary material for: Bursa-Derived Cells Show a Distinct Mechano-Response to Physiological and Pathological Loading in vitro
Source: Front Cell Dev Biol. 2021 May 31;9:657166. doi: 10.3389/fcell.2021.657166 (PMC8201779; doi:10.3389/fcell.2021.657166)
Supplement: Supplementary file 2 [file Data_Sheet_2.pdf]

frontiers

Supplementary Material

Supplementary Table 2: Surface marker expression of bursa-derived cells compared to the literature

Marker classification	Antigen	Fluorochrome	% positive cells	
			Bursa-derived cells	Bursa stem cells from literature
Mesenchymal progenitor markers	CD29	PE	99.3 (97.9-100)	
	CD44	PE-Cy7	98.7 (96.2-100)	70-95[2]; 90[3]
	CD73	APC	95.3 (85.8-100)	97.5[1]; 90[3]
	CD90	PerCP-Cy5.5	90.7 (70.2-100)	95.2[1]; 81[2]; 100[3]
	CD105	FITC	99.7 (99.1-100)	98.1[1]; 12.8[2]; 95[3]
Hematopoietic markers	CD11b	V450	1.7 (0.8-3.3)	
	CD14	PC-Cy7	1.5 (0.7-2.7)	
	CD19	PE	0.2 (0-0.6)	
	CD34	FITC	5.5 (1.2-9.3)	7.7[1]; 0[2]
	CD45	PerCP	1.2 (0.1-0.5)	0[2,3]

1) (Steinert et al. 2015); 2) (Utsunomiya et al. 2013); 3) (Morikawa et al. 2020)

References:

Morikawa, D., L. N. Muench, J. B. Baldino, C. Kia, J. Johnson, A. Otto, L. Pauzenberger, F. Dyrna, M. B. R. McCarthy, and A. D. Mazzocca. 2020. 'Comparison of Preparation Techniques for Isolating Subacromial Bursa-Derived Cells as a Potential Augment for Rotator Cuff Repair', *Arthroscopy*, 36: 80-85.

Steinert, A. F., M. Kunz, P. Prager, S. Gobel, L. Klein-Hitpass, R. Ebert, U. Noth, F. Jakob, and F. Gohlke. 2015. 'Characterization of bursa subacromialis-derived mesenchymal stem cells', *Stem Cell Res Ther*, 6: 114.

Utsunomiya, H., S. Uchida, I. Sekiya, A. Sakai, K. Moridera, and T. Nakamura. 2013. 'Isolation and characterization of human mesenchymal stem cells derived from shoulder tissues involved in rotator cuff tears', *Am J Sports Med*, 41: 657-68.
