# Supplementary material for: Bursa-Derived Cells Show a Distinct Mechano-Response to Physiological and Pathological Loading in vitro
Source: Front Cell Dev Biol. 2021 May 31;9:657166. doi: 10.3389/fcell.2021.657166 (PMC8201779; doi:10.3389/fcell.2021.657166)
Supplement: Supplementary file 3 [file Image_1.pdf]

Supplementary Material

Supplementary Figures

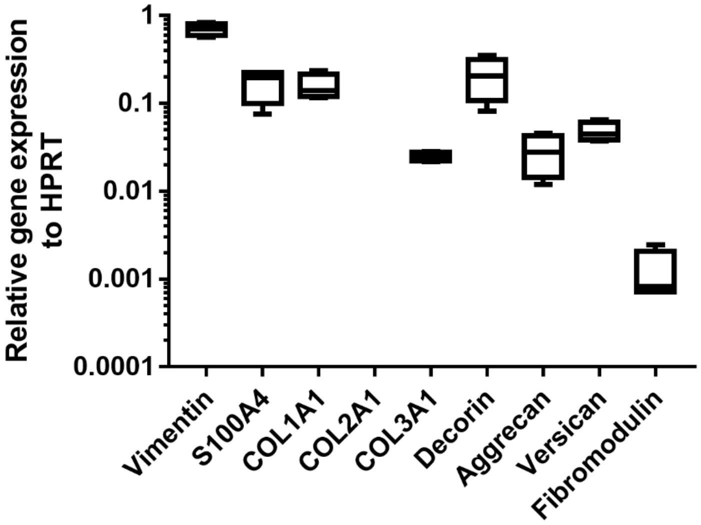

Supplementary Figure 1: Expression profile of fibroblast-associated and ECM markers of bursa-derived cells given as relative gene expression to the housekeeping gene HPRT.
