# Supplementary material for: Bursa-Derived Cells Show a Distinct Mechano-Response to Physiological and Pathological Loading in vitro
Source: Front Cell Dev Biol. 2021 May 31;9:657166. doi: 10.3389/fcell.2021.657166 (PMC8201779; doi:10.3389/fcell.2021.657166)
Supplement: Supplementary file 4 [file Image_2.pdf]

Supplementary Material

Supplementary Figures

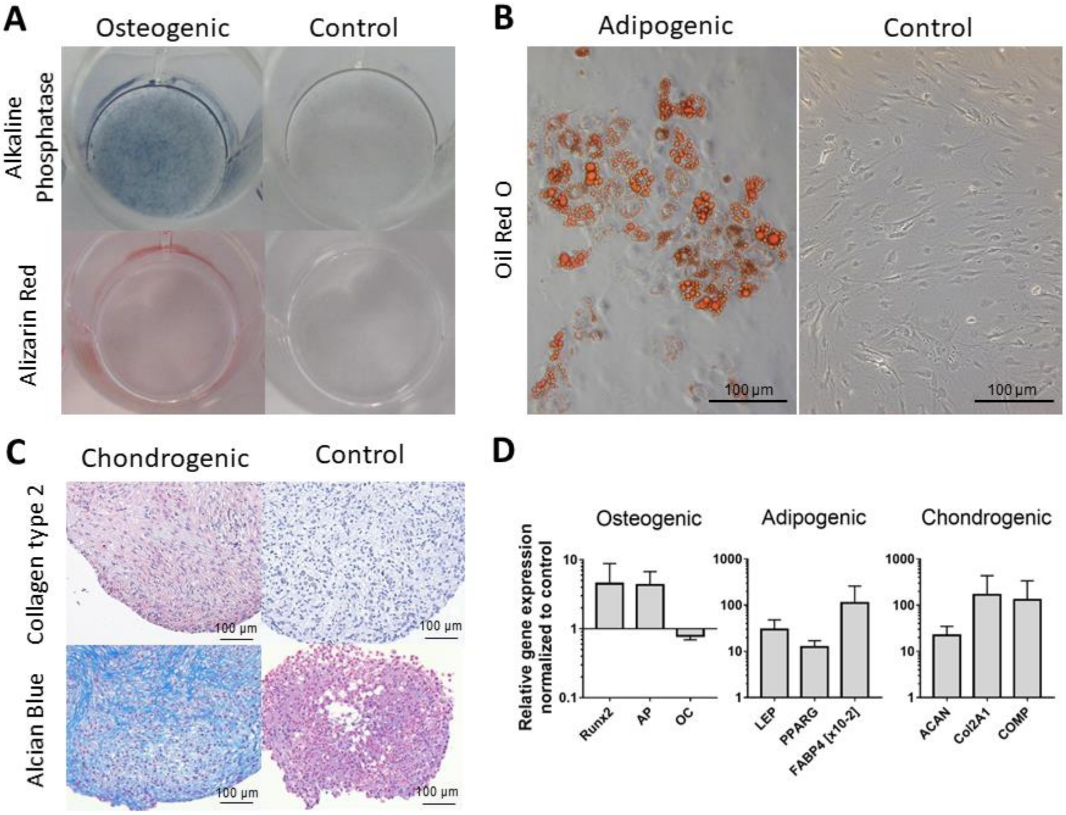

Supplementary Figure 2: Representative images of multipotent differentiation potential of bursa-derived cells. A) Alizarin red staining was relatively weak, but alkaline phosphatase staining revealed differentiation into the osteogenic direction. Macroscopic pictures of 12-well plate. B) Adipogenic differentiation visualized by Oil Red O staining of lipid vacuoles. Scale: 100 µm. C) Chondrogenic differentiated cell pellets stained for Collagen type II and Alcian Blue. Scale: 100 µm. D) Relative gene expression of osteogenic, adipogenic and chondrogenic markers normalized to 18s rRNA and given as fold to the undifferentiated control (line at 1).
